# Combined Effect of a Microporous Layer and Type I Collagen Coating on a Biphasic Calcium Phosphate Scaffold for Bone Tissue Engineering

**DOI:** 10.3390/ma8031150

**Published:** 2015-03-16

**Authors:** Mun-Hwan Lee, Changkook You, Kyo-Han Kim

**Affiliations:** 1 Department of Medical & Biological Engineering, Graduate School, Kyungpook National University, 2-188-1 Samduk-dong, Jung-gu, Daegu 700-412, Korea; E-Mails: leemunhwan@knu.ac.kr (M.-H.L.); ckyou@ynu.ac.kr (C.Y.); 2 Department of Dental Biomaterials, School of Dentistry, Kyungpook National University, 2-188-1 Samduk-dong, Jung-gu, Daegu 700-412, Korea

**Keywords:** biphasic calcium phosphate, microporous layer, type I collagen, combined effect, bone tissue engineering

## Abstract

In this study, type I collagen was coated onto unmodified and modified microporous biphasic calcium phosphate (BCP) scaffolds. Surface characterization using a scanning electron microscope (SEM) and a surface goniometer confirmed the modification of the BCP coating. The quantity of the collagen coating was investigated using Sirius Red staining, and quantitative assessment of the collagen coating showed no significant differences between the two groups. MG63 cells were used to evaluate cell proliferation and ALP activity on the modified BCP scaffolds. The modified microporous surfaces showed low contact angles and large surface areas, which enhanced cell spreading and proliferation. Coating of the BCP scaffolds with type I collagen led to enhanced cell-material interactions and improved MG63 functions, such as spreading, proliferation, and differentiation. The micropore/collagen-coated scaffold showed the highest rate of cell response. These results indicate that a combination of micropores and collagen enhances cellular function on bioengineered bone allograft tissue.

## 1. Introduction

Bone defects, including osteo-degenerative diseases, tumors, bone loss, and fractures, have great socioeconomic impact in disability [1]. Treatment of bone deficiencies remains a challenge for skeletal and orthopedic trauma surgery [2]. Autograft bone substitute, harvested from other parts of the patient’s body, are considered to be the gold standard for bone repair applications. However, autografting has several problems, such as limited procurement, potential morbidity at the donor site, and risk of wound infection, which may restrict its popular use [3]. These limitations can be overcome by the application of allografts. However, allografts pose the risk of transmission and immune diseases. Thus, the concern over their use has led to the development of synthetic bone substitutes as an alternative.

Some of the most promising ceramic synthetic bone substitutes are calcium phosphate ceramics, including hydroxyapatite (HAp), β-tricalcium phosphate (β-TCP), and biphasic calcium phosphate (BCP), which is a mixture of HAp and β-TCP. BCP benefits from the combination of the stability of HAp and the reactivity of β-TCP. The HAp component usually resolves slowly in vivo via osteoclastic resorption, because the solubility and dissolution rate of β-TCP are relatively higher than those of HAp; β-TCP therefore dissolves much faster than HAp, both in vitro and in vivo. The rapid rate of β-TCP dissolution can be retarded by combination with HAp, with harmony of new bone formation rate [4,5].

A scaffold for tissue regeneration should be similar to natural bone in both mechanical properties and structure. Additionally, scaffold architecture is a key factor in determining the rate of bone ingrowth [6,7,8]. There are two key parameters: (1) pore size and (2) pore interconnectivity. It has been demonstrated that interconnected macroporosity (diameter > 100 μm) allows blood vessel ingrowth to the pores and provides a scaffold for provision of nutrients to cells and colonization by bone cells [8]. However, many previous studies have shown that microporosity (diameter < 10 μm) improves bone regeneration by increasing the surface area available for protein adsorption [9], providing more attachment sites for osteoblasts [8], and improving ionic solubility [10,11]. Additionally, the interconnectivity of micropores has been shown to have a positive influence on the circulation of bodily fluids [12] and the bone deposition rate [8,13].

Biochemical modifications of the surface of biomaterials, inspired by the current understanding of the biology and biochemistry of cellular function, have recently been attracting much attention. Biomaterials are biochemically modified in order to induce specific cell and tissue responses by immobilizing components of the extracellular matrix (ECM), peptides, or enzymes onto the surface [14,15,16]. Type I collagen, one of the most abundant structural proteins in hard tissues, is a well-known mediator of osteoblast cellular functions, including initial attachment, proliferation, and differentiation [17,18,19]. In particular, type I collagen has successfully been used to modify the surface of biomaterials, by introducing additional bioadhesive motifs such as asparagine-glycine-glutamate-alanine (DGEA) or glycine-phenylalanine-hydroxyproline-glycine-glutamate-arginine (GFOGER), both of which are thought to be binding ligands for the α2β1 integrins of osteoblasts [20,21]. It has been reported that the collagen coatings enhance not only the biological but also the mechanical properties of ceramic scaffolds [22,23]. However, the effect of coating a microporous surface with collagen has not been reported to date.

In the present *in vitro* study, we assessed the combined effect of addition of a microporous surface and a type I collagen coating to a BCP scaffold on *in vitro* cellular behavior, for enhancement of scaffold–osteoblast interactions. The experimental groups were as follows: (a) an unmodified BCP scaffold (control); (b) an unmodified scaffold with a microporous surface layer (MP); (c) an unmodified scaffold with a type I collagen coating (COL); (d) a scaffold modified with a microporous surface layer and a type I collagen coating (MP/COL).

## 2. Results and Discussion

### 2.1. Surface Characterization

**Figure 1:**
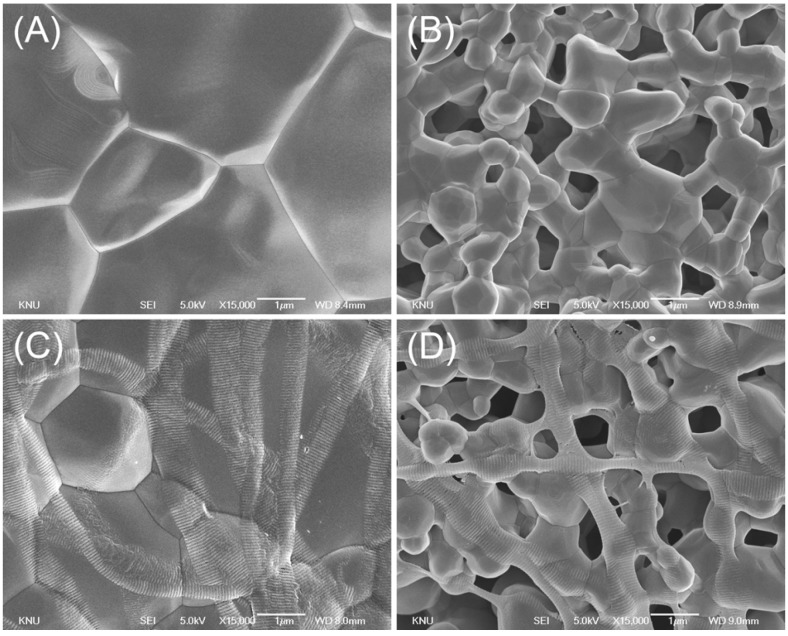
SEM images of scaffold microstructure (15,000×, bar = 1 μm); (**A**) Control; (**B**) micropore (MP); (**C**) collagen (COL); (**D**) micropore/collagen-combined (MP/COL).

In Figure 1, the SEM images of the non-collagen-coated microstructure clearly show two different surface structures (smooth and microporous surfaces) (Figure 1A,B). In the control group, the surface was smooth, with clearly demarcated grain boundaries. In contrast, for the micropore-modified group (group MP), the microporous layer was formed on each strut of the scaffold (Figure 1B) via aggregation of BCP particles. Pseudo-physiological/biological treatment increases the microporosity of calcium phosphate scaffold [24], but such increasing may reduce the stiffness and strength of the scaffold [25,26]. In this study, however, the bulk property of the scaffold was not changed because the microporous layer was coated on the surface. For the collagen-coated group (COL), the scaffold surface of the strut was homogeneously covered by collagen fibers. In the micropore/collagen-combined group (MP/COL) the microporous layer could still be observed.

### 2.2. Surface Wettability

**Figure 2:**
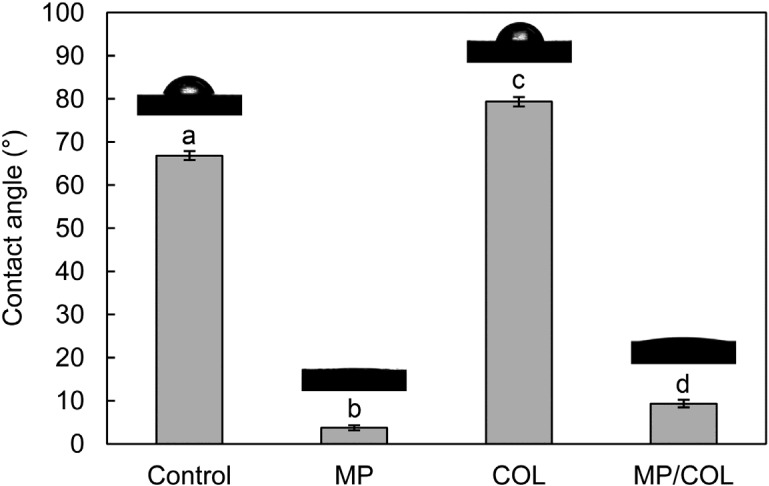
Contact angles of a drop of water on the four different surfaces. Identical lower-case letters indicate statistically equivalent values (*p* > 0.05).

The surface hydrophilicity is a key factor that determines the response of cells to biomaterials. The CA is the angle where a liquid/vapor interface meets a solid surface, and it is dependent on the surface area. Furthermore, a lower CA indicates a higher surface energy. The microporous surface of the BCP scaffold encourages liquids to sink into the pores, and thus improves surface wettability by decreasing the CA [21,27]. Figure 2 shows the water CA values for the four groups. The CA value of the control group was 66.9° ± 1.6°. In the MP group, the CA values were dramatically lower, at 3.8° ± 0.6°. The COL group showed the highest CA (79.3° ± 1.1°), the difference being statistically significant (*p* < 0.05). The CA value for the MP/COL group was also low, at 9.4° ± 0.9°. In this study, therefore, the total surface energy of the microporous surfaces was higher than that of the smooth surfaces. The collagen-coated surface showed a slightly higher CA than the collagen-free surfaces. This result indicates that the collagen coating reduced wettability. However, the CA of the micropore/collagen-combined surface was markedly lower, at 9.4° ± 0.9°. Although the microporous layers were partially covered by collagen, the basic surface properties of the microporous layers remained.

### 2.3. Quantitative Examination of the Type I Collagen Coatings

**Figure 3:**
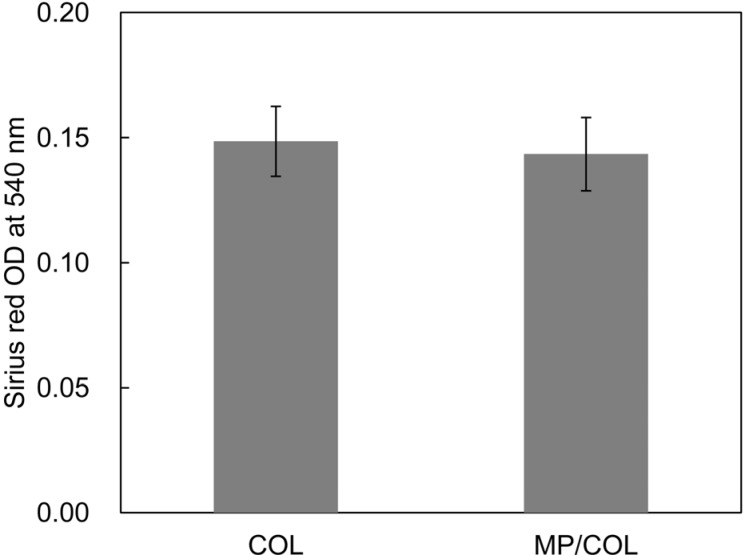
The amount of type I collagen on the surfaces.

Collagen stained with Sirius Red was observed in groups COL and MP/COL (Figure 3). Quantitative assessment of the collagen coating indicated that there were no significant differences between the two groups. This finding suggests that the quantity of collagen adsorption on the scaffold surfaces was not dependent on the surface microstructure under these experimental conditions.

### 2.4. Cell Morphology

**Figure 4:**
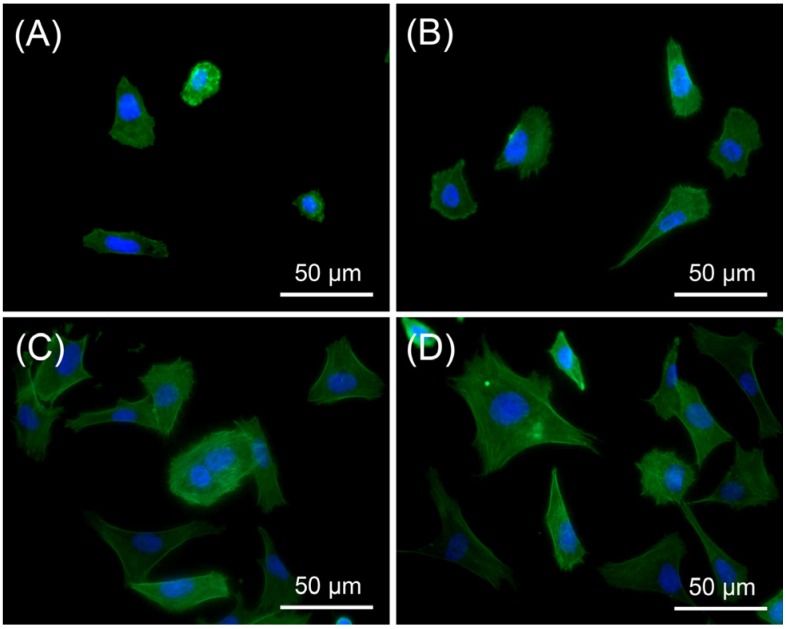
Fluorescent microscopy images of MG63 cells on (**A**) Control; (**B**) MP; (**C**) COL; (**D**) MP/COL (actin cytoskeleton (green)—Alexa 488, nucleus (blue)—DAPI).

**Figure 5:**
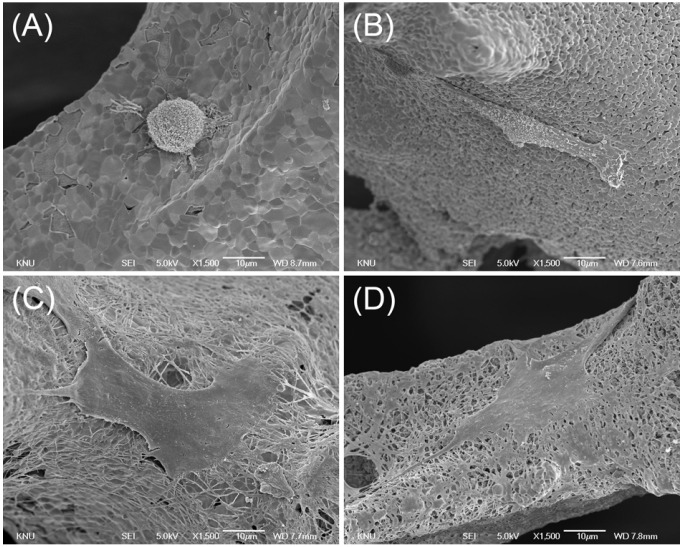
SEM images of MG63 cells cultured on (**A**) Control; (**B**) MP; (**C**) COL; (**D**) MP/COL (1,500×, bar = 10 μm).

In this study, the initial interaction between osteoblasts and the surfaces was found to be correlated with the CA values. As shown in Figure 4 and Figure 5, cells on the smooth surfaces were round, whereas on the microporous surfaces they were more elongated. This finding indicates that the microporous surface was a more biocompatible environment for osteoblast adhesion than the smooth surface. The collagen-coated surfaces showed higher surface CA values, but showed greater spreading than the collagen-free surfaces. Coating with type I collagen successfully modified the surfaces by addition of bioadhesive motifs such as asparagine-glycine-glutamate-alanine (DGEA) or glycine-phenylalanine-hydroxyproline-glycine-glutamate-arginine (GFOGER). These motifs are known to be binding ligands for the α2β1 integrins of osteoblasts [20,28]. In the collagen-free groups, surface wettability affected the initial adhesion of the MG63 cells. The results for the two collagen-coated groups suggest that the biochemical effect was greater than the micropore effect for promoting initial cell adhesion.

### 2.5. Cell Proliferation

**Figure 6:**
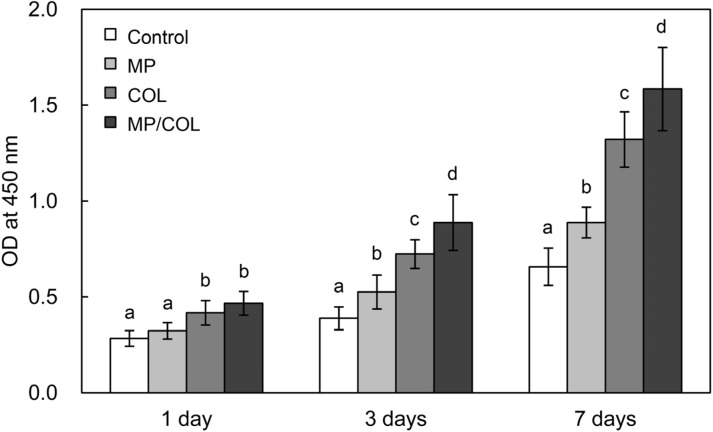
Proliferation of MG63 cells on the four experimental surfaces.

The relationship between a cell and a biomaterial may influence the response of osteoblast cells to migrate to the surface of the material [29]. To determine the effect of the microporous layer and the collagen coating on MG63 cell proliferation, the number of cells on each surface was estimated using a WST assay at days 1, 3, and 7. In all groups, the number of cells increased significantly with culture time (Figure 6). At day 1, the number of cells on the COL and MP/COL scaffolds was significantly higher than on the control scaffolds. In contrast, no significant difference was observed between the control group and the MP group. At day 3 and 7, the number of cells was significantly higher on all treated groups than on the control group. Cell proliferation was significantly higher in the COL group than in the MP group (*p* < 0.05). In particular, the MP/COL scaffolds showed the highest proliferation value after 3 days of cell culture, suggesting the effective combination of physical effect of the micropores and the biochemical effect of collagen.

### 2.6. ALP Activity

**Figure 7:**
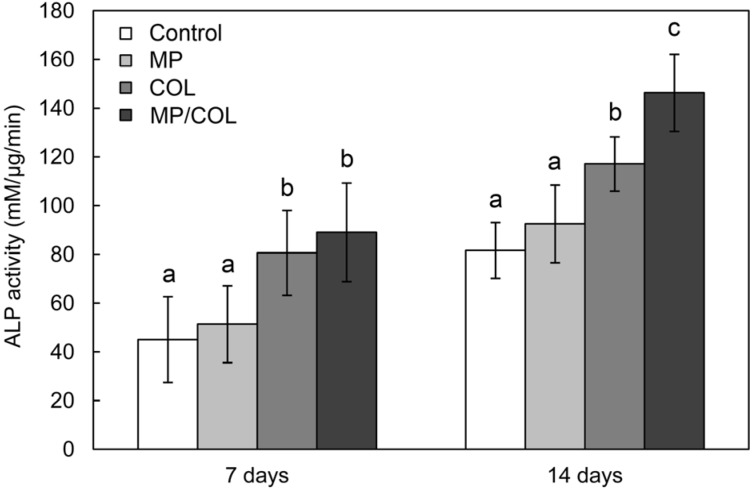
ALP activity of MG63 cells on the four experimental surfaces.

Alkaline phosphatase activity (ALP) is an early-stage marker of osteoblast differentiation [30]. Previous studies have shown that surfaces with rougher textures promote osteoblast differentiation [21,31,32]. No significant differences in ALP activity were observed between the cells growing on the control scaffold and the MP scaffold. ALP activity was significantly higher in cells grown on the collagen-coated surfaces than in cells grown on the collagen-free surfaces. These results indicate that the collagen surface coating encouraged osteogenic differentiation of MG63 cells. The collagen molecules on the scaffold surface were similar to the native structure of the ECM. The enhanced cell spreading might result in earlier and stronger osteogenic differentiation by triggering the adhesion-induced signaling pathway [33]. Furthermore, the MP/COL group showed higher ALP activity than the collagen-coated smooth surface, indicating enhanced osteoinduction of adherent cells after 14 days of cell culture. The findings of this in vitro study suggest that combining a microporous layer and a type I collagen coating on BCP scaffolds produces a dramatically improved biomaterial (Figure 7).

## 3. Experimental Section

### 3.1. Preparation of the Unmodified Scaffolds

Polyurethane (PU) sponge was treated in 2 wt% of NaOH solution for 10 min and dried slowly at 60 °C after washing with distilled water. The scaffolds were prepared using PU sponges infiltrated with ceramic slurry. For preparation of the primary slurry, biphasic calcium phosphate (BCP) powder was mixed and kneaded with 3 wt% polyvinyl alcohol (PVA) (Sigma-Aldrich Co., St. Louis, MO, USA) solution at a ratio of 1.5:1 by weight. The primary coating slurry had a high viscosity to produce a smooth surface. The PU sponge was placed in the BCP slurry and rolled with a rod, followed by repeated compression/release for fabrication of the scaffold. Then, the BCP-coated PU sponge was dried at room temperature for 12 h and sintered at 1200 °C for 3 h.

Disc-shaped BCP specimens (10 mm in diameter and 2 mm in height), which were subjected to the same manufacturing and surface modification process as the scaffold, were used to assess the surface contact angle and for fluorescent imaging of MG63 morphology.

### 3.2. Surface Modification of the Scaffolds

As stated earlier, four groups of experimental samples were prepared, as follows: (1) control; (2) MP; (3) COL; and (4) MP/COL.

For the MP group, a microporous layer was prepared using the BCP slurry dipping method. The low-viscosity BCP slurry was prepared by dispersing BCP powder in 3 wt% PVA solution at a ratio of 1:3 by weight. The BCP scaffold was dipped in the slurry and blown by dried air, to eliminate excess slurry from the specimens. The specimens were dried at room temperature for 6 h and sintered at 1150 °C for 2 h.

For the COL group, type I collagen (BNC KOREA, Daegu, Korea) extracted from bovine Achilles tendon was dissolved in 2 mM hydrochloric acid at a concentration of 0.0125 wt%. Prepared specimens were soaked in the collagen suspension for 10 min, and cleaned ultrasonically with water and then finally dried under vacuum.

### 3.3. SEM Examination

The BCP scaffold materials that had been subjected to the surface modification processes were examined using a field emission-scanning electron microscope (FE-SEM, JSM-6700F, Jeol, Tokyo, Japan). The surface morphology of each group was evaluated under a FE-SEM at 15,000× magnification after platinum sputter coating.

### 3.4. Surface Wettability

The contact angles (CAs) of water droplets on the disc-shaped BCP surfaces were measured using the static sessile drop method with a surface goniometer (OCA 15 plus, Data-Physics Instrument GmbH, Filderstadt, Germany).

### 3.5. Quantitative Examination of Collagen Coating

Sirius Red staining, which binds specifically to collagen fibrils, is used for examination of collagen [28]. All experimental groups were immersed in 1 mg/mL Picro-Sirius Red F3B dye (Klinipath, Geel, Belgium) for 30 min. After rinsing in distilled water, the dyed specimens were dried. To dissolve the dye, the specimens were immersed in 0.5 M NaOH solution for 2 h. The absorbance of the extraction solutions was assessed at 540 nm using a UV/VIS-spectrophotometer (UV-1600PC, Shimadzu, Tokyo, Japan). To quantify the amount of the coated collagen alone, the absorbance values of groups control and MP were subtracted from those of groups COL and MP/COL.

### 3.6. Cell Culture

Each scaffold (10 mm in diameter and 3 mm in height) was sterilized by gamma irradiation. Human pre-osteoblastic cells, of the MG63 cell line, were cultured in alpha minimum essential medium (α-MEM) containing 1 g/L glucose supplemented with 10% fetal bovine serum (FBS) and 1% penicillin/streptomycin in a humidified incubator (37 °C and 5% CO_2_). Prior to cell seeding, the scaffold materials were placed in 24-well plates and incubated with the medium for 2 h. The MG63 cells (3 × 10^4^ cells per scaffold) were seeded onto the top of the scaffold after removed the medium completely. The 24-well plate was left in the incubator for 2 h to allow the MG63 cells to adhere to the surface. Thereafter, 1 mL of additional medium was added to each well.

### 3.7. Cell Morphology

MG63 cells on the scaffolds were fixed in 2.5% glutaraldehyde, postfixed in 1% osmium tetroxide, and then dehydrated using a graded ethanol series. After the dehydration step, the scaffolds were immersed in a mixture of 100% ethanol and isoamyl acetate (1:1, v/v) and then in absolute isoamyl acetate. The cellular morphology on the surfaces was observed by SEM after platinum sputter coating. For fluorescence imaging, the specimens were stained with different fluorescent dyes. 4′,6-Diamidino-2-phenylindol (DAPI) was used to stain cell nuclei. Alexa Fluor^®^ 488 Phalloidin (Eugene, OR, USA) was used to stain the actin cytoskeletons. The specimens were removed from the 24-well plates, rinsed three times in PBS, and the cells were fixed for 15 min in a 3.7% formaldehyde solution. The cells fixed on the specimens were then rinsed and permeabilized using a 0.5% Triton X-100 solution (BDH Laboratories, Poole, UK) for 15 min. Specimens were then washed three times with PBS and stained to visualize F-actin and nucleic acids. Fluorescence images were collected with a fluorescent microscope (BX53, Olympus, Center Valley, PA, USA).

### 3.8. Cell Proliferation

The proliferation of MG63 cells on the experimental materials was assessed using a Cell Counting Kit-8 (CCK-8; Dojindo, Tokyo, Japan). In this assay, 2-(2-methoxy-4-nitrophenyl)-3-(4-nitrophenyl)-5-(2,4-disulfophenyl)-2H-tetrazolium, monosodium salt (WST-8) is reduced by dehydrogenases in the cells to produce an orange-colored product (formazan) that is soluble in the tissue culture medium. The amount of the formazan dye generated by the dehydrogenases in the cells is directly proportional to the number of living cells. After 1, 3, and 7 days of culture, the scaffold was transferred to new plates and washed with PBS. Then 850 µL of culture medium and 50 µL of WST-8 from the labeling kit were added. To assess the absorbance using a Sunrise^®^ microplate reader (Tecan Austria GmbH, Grödig, Austria), 200 μL of the suspension was transferred to a 96-well culture plate. The absorbance of the solution was evaluated at a wavelength of 450 nm.

### 3.9. ALP Activity

Cell differentiation was evaluated using an early marker of osteoblast differentiation: cellular alkaline phosphatase activity. ALP activity was assayed by measuring the release of *p*–nitrophenol from *p*–nitrophenylphosphate at pH 10.2. Activity values were normalized to the protein content, which was detected as colorimetric cuprous cations in a biuret reaction (BCA Protein Assay Kit, Pierce Biotechnology Inc., Rockford, IL, USA) at 570 nm. The optical density was measured using a Sunrise^®^ microplate reader.

### 3.10. Statistical Analysis

All experiment were performed in triplicate and the results are presented as mean ± standard deviation. The results were analyzed using a one-way ANOVA. Differences were considered significant if *p* < 0.05.

## 4. Conclusions

In this experiment, BCP scaffolds were successfully modified using a BCP slurry dipping method and collagen adsorption coating. Addition of a microporous layer improved surface wettability and coating with type I collagen enhanced osteoblast cells’ interaction with integrin. Microporous layer modification, collagen coating, and the combination of the two on a BCP scaffold enhanced osteoblast cell response (spreading, proliferation, and differentiation). In particular, the micropore/collagen-coated scaffold showed the highest rate of cell response. These results indicate a combined effect of micropores and collagen in terms of cellular functions.

## References

[1] Van Lieshout E.M.M., van Kralingen G.H., El-Massoudi Y., Weinans H., Patka P. (2011). Microstructure and biomechanical characteristics of bone substitutes for trauma and orthopaedic surgery. Bmc Musculoskel. Dis..

[2] Wojtowicz A.M., Shekaran A., Oest M.E. (2010). Coating of biomaterial scaffolds with the collagen-mimetic peptide GFOGER for bone defect repair. Biomaterials.

[3] De Long W.G., Einhorn T.A., Koval K. (2007). Bone, grafts and bone graft substitutes in orthopedic trauma surgery—A critical analysis. J. Bone Joint Surg. Am..

[4] Li S.H., de Wijn J.R., Li J.P., Layrolle P., de Groot K. (2003). Macroporous biphasic calcium phosphate scaffold with high permeability/porosity ratio. Tissue Eng..

[5] Ellinger R.F., Nery E.B., Lynch K.L. (1986). Histological assessment of periodontal osseous defects following implantation of hydroxyapatite and biphasic calcium phosphate ceramics: A case report. Int. J. Periodontics Restor. Dent..

[6] Jin Q.M., Takita H., Kohgo T., Atsumi K., Itoh H., Kuboki Y. (2000). Effects of geometry of hydroxyapatite as a cell substratum in BMP-induced ectopic bone formation. J. Biomed. Mater. Res..

[7] Kaito T., Myoui A., Takaoka K. (2005). Potentiation of the activity of bone morphogenetic protein-2 in bone regeneration by a PLA-PEG/hydroxyapatite composite. Biomaterials.

[8] Bignon A., Chouteau J., Chevalier J. (2003). Effect of micro- and macroporosity of bone substitutes on their mechanical properties and cellular response. J. Mater. Sci. Mater. Med..

[9] Hing K.A., Annaz B., Saeed S., Revell P.A., Buckland T. (2005). Microporosity enhances bioactivity of synthetic bone graft substitutes. J. Mater. Sci. Mater. Med.

[10] Habibovic P., Yuan H.P., van der Valk C.M., Meijer G., van Blitterswijk C.A., de Groot K. (2005). 3D microenvironment as essential element for osteoinduction by biomaterials. Biomaterials.

[11] Le Nihouannen D., Daculsi G., Saffarzadeh A. (2005). Ectopic bone formation by microporous calcium phosphate ceramic particles in sheep muscles. Bone.

[12] Legeros R.Z., Lin S., Rohanizadeh R., Mijares D., Legeros J.P. (2003). Biphasic calcium phosphate bioceramics: Preparation, properties and applications. J. Mater. Sci. Mater. Med..

[13] Hing K.A., Best S.M., Tanner K.E., Bonfield W., Revell P.A. (2004). Mediation of bone ingrowth in porous hydroxyapatite bone graft substitutes. J. Biomed. Mater. Res. A.

[14] Puleo D.A., Nanci A. (1999). Understanding and controlling the bone-implant interface. Biomaterials.

[15] Sousa I., Mendes A., Pereira R.F., Bartolo P.J. (2014). Collagen surface modified poly (ε-caprolactone) scaffolds with improved hydrophilicity and cell adhesion properties. Mater. Lett..

[16] Nie L., Chen D., Fu J., Yang S., Hou R., Suo J. (2015). Macroporous biphasic calcium phosphate scaffolds reinforced by poly-L-lactic acid/hydroxyapatite nanocomposite coatings forbone regeneration. Biochem. Eng. J..

[17] Takeuchi Y., Nakayama K., Matsumoto T. (1996). Differentiation and cell surface expression of transforming growth factor-beta receptors are regulated by interaction with matrix collagen in murine osteoblastic cells. J. Biol. Chem..

[18] Mizuno M., Fujisawa R., Kuboki Y. (2000). Type I collagen-induced osteoblastic differentiation of bone-marrow cells mediated by collagen-α_2_β_1_ integrin interaction. J. Cell. Physiol..

[19] Roehlecke C., Witt M., Kasper M. (2001). Synergistic effect of titanium alloy and collagen type I on cell adhesion, proliferation and differentiation of osteoblast-like cells. Cells Tissues Organs.

[20] Reyes C.D., Garcia A.J. (2003). Engineering integrin-specific surfaces with a triple-helical collagen-mimetic peptide. J. Biomed. Mater. Res. A.

[21] Zhang Y.M., Bataillon-Linez P., Huang P. (2004). Surface analyses of micro-arc oxidized and hydrothermally treated titanium and effect on osteoblast behavior. J. Biomed. Mater. Res. A.

[22] Li W., Pastrama M.I., Ding Y., Zheng K., Hellmich C., Boccaccini A.R. (2014). Ultrasonic elasticity determination of 45S5 Bioglass^®^-based scaffolds: Influence of polymer coating and crosslinking treatment. J. Mech. Behav. Biomed. Mater..

[23] Hum J., Luczynski K.W., Nooeaid P., Newby P., Lahayne O., Hellmich C., Boccaccini A.R. (2013). Stiffness improvement of 45S5 Bioglass^®^-based scaffolds through natural and synthetic biopolymer coatings: An ultrasonic study. Strain.

[24] Czenek A., Blanchard R., Dejaco A., Sigurjonsson O.E., Orlygsson G., Gargiulo P. (2014). Quantitative intravoxel analysis of μCT-scanned resorbing ceramic biomaterials—Perspectives for computer-aided biomaterial design. J. Mater. Res..

[25] Fritsch A., Dormieux L., Hellmich C., Sanahuja J. (2009). Mechanical behavior of hydroxyapatite biomaterials: An experimentally validated micromechanical model for elasticity and strength. J. Biomed. Mater. Res. A.

[26] Fritsch A., Hellmich C., Young P. (2013). Micromechanics-derived scaling relations for poroelasticity and strength of brittle porous polycrystals. J. Appl. Mech..

[27] Ye X.Y., Cai S., Xu G.H., Dou Y., Hu H.T., Ye X.J. (2013). Preparation and in vitro evaluation of mesoporous hydroxyapatite coated β-TCP porous scaffolds. Mater. Sci. Eng. C.

[28] Muller R., Abke J., Schnell E. (2006). Influence of surface pretreatment of titanium- and cobalt-based biomaterials on covalent immobilization of fibrillar collagen. Biomaterials.

[29] Suh H., Hwang Y.S., Lee J.E., Han C.D., Park J.C. (2001). Behavior of osteoblasts on a type I atelocollagen grafted ozone oxidized poly L-lactic acid membrane. Biomaterials.

[30] Holtorf H.L., Jansen J.A., Mikos A.G. (2005). Ectopic bone formation in rat marrow stromal cell/titanium fiber mesh scaffold constructs: Effect of initial cell phenotype. Biomaterials.

[31] Takeuchi K., Saruwatari L., Nakamura H.K., Yang J.M., Ogawa T. (2005). Enhanced intrinsic biomechanical properties of osteoblastic mineralized tissue on roughened titanium surface. J. Biomed. Mater. Res. A.

[32] Ogawa T., Ozawa S., Shih J.H. (2000). Biomechanical evaluation of osseous implants having different surface topographies in rats. J. Dent. Res..

[33] Lee Y.J., Ko J.S., Kim H.M. (2006). The role of cell signaling defects on the proliferation of osteoblasts on the calcium phosphate apatite thin film. Biomaterials.

